# Upper Urinary Tract Urothelial Cancer After Radical Cystectomy for Bladder Cancer: Survival Outcomes After Radical Nephroureterectomy

**DOI:** 10.1245/s10434-023-14710-x

**Published:** 2023-12-12

**Authors:** Aleksander Ślusarczyk, Piotr Zapała, Tomasz Piecha, Łukasz Zapała, Tomasz Borkowski, Piotr Radziszewski

**Affiliations:** Department of General, Oncological and Functional Urology, Medical University of Warsaw, Warsaw, Poland

**Keywords:** Radical nephroureterectomy, Radical cystectomy, Bladder cancer, Upper urinary tract urothelial cancer, Urothelial cancer, Survival

## Abstract

**Background:**

Systemic and local recurrences of urothelial bladder cancer (UBC) significantly impair survival after radical cystectomy (RC), but little is known about the impact of the recurrence of urothelial cancer in the upper urinary tract (UTUC). This report describes survival outcomes and their predictors for patients who underwent RC followed by radical nephroureterectomy (RNU) for UTUC.

**Methods:**

The Surveillance, Epidemiology, and End Results database was queried to identify patients who underwent RC for UBC and subsequent RNU for UTUC. The Kaplan–Meier method and competing-risk Cox regression (CRR) were used for the survival analysis.

**Results:**

Overall, 102 patients have undergone RNU within a median of 49 months (interquartile range [IQR], 27–76 months) since RC. Muscle-invasive UTUCs were predominant at RNU (*n* = 58; 56.7%), but organ-confined bladder tumors were most frequent at RC (*n* = 42, 41.5%). After RNU, the estimated 5-year overall survival (OS) was 25.9%, the cancer-specific survival (CSS) was 35.6%, the median OS was 23 months (IQR, 11–63 months), and the CSS was 34 months (IQR, 13–132 months). In the multivariable CRR, the factors predictive for CSS after RNU included male gender (hazard ratio [HR], 2.36; 95% confidence interval [CI], 1.03–5.42; *p* < 0.05), muscle-invasive UTUC (HR, 2.20; 95% CI, 1.13–4.28; *p* < 0.05), and the presence of distant metastasis (HR,11.59; 95% CI, 5.33–25.2; *p* < 0.001).

**Conclusions:**

In conclusion, the patients who underwent RNU for UTUC after RC for UBC experienced poor OS and CSS. The majority of RNUs were performed for locally advanced tumors. The independent risk factors for worse OS and CSS after RNU were UTUC T stage, presence of metastasis, and male gender.

Bladder cancer constitutes a major risk factor for the development of upper urinary tract urothelial cancer (UTUC).^1^ Radical cystectomy (RC) remains a mainstay surgical treatment for muscle-invasive bladder cancer (MIBC) and for selected patients with high-risk non-muscle-invasive bladder cancer (NMIBC).^2,3^ Obviously, patients who undergo RC are at greater risk for UTUC than the general population.^4,5^

The estimated frequency of UTUC in RC cohorts is about 3%, although it is reported to range from 0.75% to 6.4% in different studies.^5,6^ In such patients, radical nephroureterectomy (RNU) due to urothelial cancer (UC) recurrence in the upper urinary tract (UUT) should be strongly considered, particularly for high-risk tumors. However, RNU after RC, might be especially challenging considering the burden of comorbidities and anatomic abnormalities contributing to the increased complexity of surgery. Due to the high competing mortality, RNU seems to be rarely chosen for UTUC management of these patients.^4^ A review of contemporary UTUCs in the post-RC follow-up evaluation reported that only 61% of such patients underwent RNU.^5^

Information about survival outcomes of RNU after RC is derived from small retrospective cohorts.^7,8^ One of these cohorts, consisting of 34 patients, reported a 5-year cancer-specific survival (CSS) of 45.6%, and another cohort with 64 patients showed a median overall survival (OS) of 3.1 years after RNU.^7,8^

The reported incidence of UTUC after RC seems to be lower than expected considering the field-effect theory of urothelial carcinogenesis and lower than that reported for high-risk NMIBC treated with Bacillus Calmette-Guerin (BCG) therapy (from 7.5% to even 25% within a 15-year follow-up period).^9,10^ Such discrepancy might be attributable to the high competing risk of other events, leading to a substantial mortality burden after RC. Importantly, early morbidity after RC due to gastrointestinal, infectious, and cardiovascular complications is relatively high, and the perioperative mortality rate is about 3%.^11-13^

Rapid bladder cancer recurrence and progression are other significant factors contributing to increased early mortality after RC.^12^ The majority of metastatic progressions after RC occur within 2 years after surgery, especially in the case of locally advanced bladder tumors.^4^ On the other hand, long-term survivors after RC represent a group with a substantial risk for metachronous UTUC.^14,15^

In this study, we aimed to report survival outcomes of RNU after RC and identify their predictors.

## Methods

The National Cancer Institute Surveillance, Epidemiology, and End Results (SEER) database was queried to identify patients with bladder cancer (International Classification of Diseases [ICD]-10 codes C67.0–C67.9) and subsequent UTUC (ICD-10 codes C65.9–C66.9) treated surgically between 2004 and 2019. We included patients who underwent RC for bladder cancer (procedure codes 50–80) and subsequent RNU for primary metachronous UTUC (procedure codes 40–50) using the “site-specific surgery of primary-site code” variable. Exceptionally, to assess the impact of RNU on survival among cystectomized patients, we included an additional risk-matched cohort of RC patients solely for survival comparison using the Kaplan–Meier method.

**Fig. 1:**
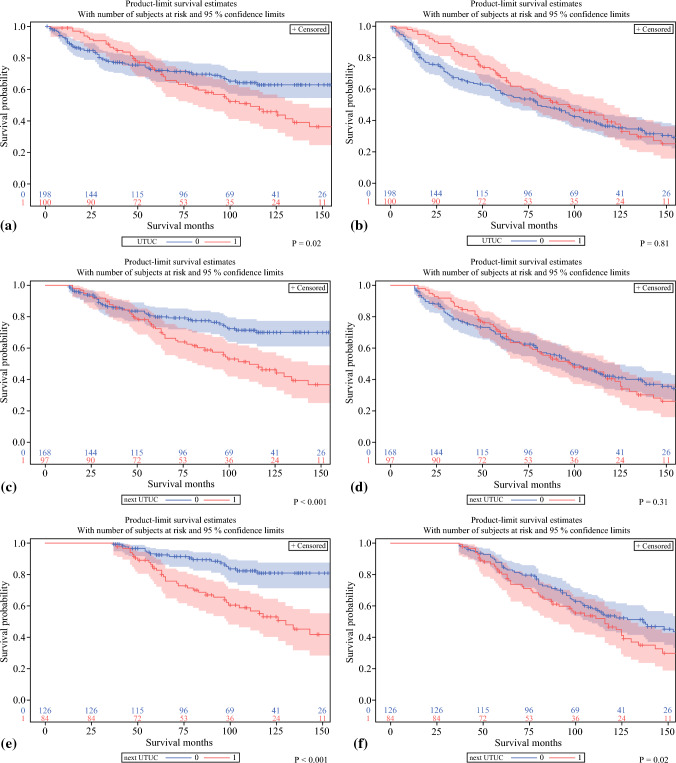
Comparison of **a** cancer-specific survival and **b** overall survival between propensity score-matched patients who underwent RNU for metachronous UTUC (red curve) after RC and their counterparts treated with RC alone (blue curve). Kaplan–Meier curves depict conditional cancer-specific survival from the landmark points of **c** 12 and **e** 36 months since RC performance. Kaplan–Meier curves depict conditional overall survival from the landmark points of **d** 12 and **f** 36 months since RC performance. RNU, radical nephroureterectomy; UTUC, urinary tract urothelial cancer; RC, radical cystectomy

The available data included information on the patient’s demographics as well as histopathologic and clinical characteristics of bladder cancer and UTUC from the time of RC and RNU, respectively. Generally, survival was calculated from the time of RNU. Cancer-specific survival was defined as the interval between UTUC diagnosis and death due to UC. Overall survival was defined as the interval between UTUC diagnosis and death due to any cause. Exceptionally, in the comparison of bladder cancer patients treated with RC between those who did and those who did not experience UTUC (treated with RNU), we calculated the survival time starting from the bladder cancer diagnosis (Fig. 1).

### Ethics

The institutional review board waived the need for study approval. The study was performed in accordance with the Declaration of Helsinki and its later amendments.

### Statistical Analysis

The Kaplan–Meier method and log-rank tests were used in survival analyses. The median OS and CSS, as well as survival rates, were estimated using the Kaplan–Meier method. Kaplan Meier curves with 95% confidence intervals (CIs) were used to illustrate survival. Conditional survival was analyzed using a landmark approach based on Kaplan–Meier estimates and calculated for patients alive 12 and 36 months after cystectomy (Fig. 1C–F). Cox proportional hazards (CPHs) were used for the prediction of survival outcomes in uni- and multivariable analyses. Competing risk regression (CRR) with CPH was performed to identify the risk factors for CSS. Stepwise selection of variables was used for multivariable analyses. Only statistically significant variables entered the final multivariable model. Hazard ratio (HR) supplemented with a 95% CI were derived from CPH and CRR. Two-sided *p* values lower than 0.05 were considered as statistically significant. Statistical analyses were performed with SAS software version 9.4.

### Subgroup Analysis to Evaluate the Impact of RNU on Survival of RC Patients

To examine the effect of RNU on survival among cystectomized patients, propensity score (PS)-matching was performed to adjust for confounders such as patient’s age, gender, race, bladder cancer T stage and grade, bladder cancer N and M stages at RC, lymph node dissection (LND) during RC, use of perioperative chemotherapy, and year of bladder cancer diagnosis. Bladder cancer patients who received RNU for UTUC after RC were PS-matched in the proportion of 1:2 with those who did not.

## Results

The study included 102 patients who underwent RC with subsequent RNU. The median follow-up period after RC was 91 months (interquartile range [IQR], 50–158 months). The majority of the patients were males (*n* = 82, 80.4%) who were younger than 70 years at the time of their RC (*n* = 59, 57.8%). Of the 102 patients, 92 were white (90.2%), only 4 (3.9%) were black, and 6 (5.9%) were of other races.

### Stages of Bladder Cancer and UTUC at the Radical Surgery

At the time of RC, 42 patients (41.2%) had a diagnosis of NMIBC, and 41 patients (40.2%) had a diagnosis of MIBC. There was a strong predominance of high-grade urothelial bladder cancer (*n* = 75, 73.5%). Lymph node (LN) dissection during RC was performed for 88 patients (87.1%), and positive LNs were diagnosed in 6 patients (5.9%). The types of urinary diversion included an ileal conduit in 32 patients (31.4%), an orthotopic neobladder in 1 patient (1%), an abdominal pouch in 1 patient (1%), and a nonspecified type in the remaining 70 patients (66.6%).

Importantly, at RNU, the muscle-invasive stages of UTUC (MIUC) (*n* = 58, 56.7%) were significantly more common than the non-muscle-invasive stages of UTUC (NMIUC) (*n* = 34, 33.3%). Of the 102 patients, 70 (68.3%) had high-grade UTUCs and 32 (31.7%) had low-grade UTUCs. The UC originated from the renal pelvicalyceal system in 68 patients (67%) and from the ureter in 34 patients (33%). During RNU, LND was performed in 49 patients (48.5%). The findings showed 63 patients (61.8%) who were LN-negative, and 23 patients (22.6%) who were LN-positive (N1–N2). Distant metastases were diagnosed in 3 patients (2.9%) at the time of RC and 6 patients (5.9%) at the time of RNU. All baseline characteristics of the patients at the time of RC and RNU were presented in Table 1.

**Table 1:** Baseline characteristics of patients at the time of radical cystectomy (RC) for bladder cancer and at subsequent radical nephroureterectomy (RNU) for upper urinary tract urothelial cancer

	Characteristics at the time of RC		Characteristics at the time of RNU	
	No. of patients	% of patients	No. of patients	% of patients
Age (years)				
<70	59	57.84	44	43.14
≥70	43	42.16	58	56.86
Gender				
Female	20	19.61	20	19.61
Male	82	80.39	82	80.39
T stage				
Tis	6	5.88	4	3.92
Ta	9	8.82	11	10.78
T1	27	26.47	19	18.63
T2	20	19.61	9	8.82
T3	9	8.82	44	43.14
T4	12	11.76	5	4.90
Unknown	19	18.63	10	9.80
N stage				
N0	77	75.49	63	61.76
N1	2	1.96	14	13.73
N2	4	3.92	9	8.82
Nx	19	18.63	16	15.69
Metastasis				
M0	79	77.45	91	89.22
M1	3	2.94	6	5.88
Mx	20	19.61	5	4.90
Grade				
Low	27	26.47	32	31.37
High	75	73.53	70	68.63
Tumor size (cm)				
≤2	12	11.76	18	17.65
>2	30	29.41	58	56.86
Unknown	60	58.82	26	25.49
Chemotherapy				
No/unknown	81	79.41	83	81.37
Yes	21	20.59	19	18.63
Lymphadenectomy				
No	13	12.87	52	51.49
Yes	88	87.13	49	48.51
Tumor location				
Renal pelvis	–	–	68	66.67
Ureter	–	–	34	33.33
Laterality				
Right	–	–	50	49.02
Left	–	–	52	50.98

### RNU Timing and Survival Outcomes

Due to the UTUC diagnosis, RNU was performed within a median of 49 months (IQR, 27–76 months) after RC. In the majority of the patients, UTUC was developed late after RC, and in only 37 individuals (36.3%) UTUC occured within 3 years after RC. Early perioperative 3-month all-cause mortality after RNU was 10.4% (*n* = 10).

Estimated survivals after RNU included a 2-year OS of 50.0%, a 5-year OS of 25.9%, a 2-year CSS of 56.2%, and a 5-year CSS of 35.6%. After RNU, the median OS was 23 months (IQR, 11–63 months), and the CSS was 34 months (IQR, 13–132 months).

### Univariable Analyses for CSS and OS After RNU

In univariable competing-risk Cox regression analyses, the following factors were associated with CSS: age of 70 years or older at RC (HR, 1.95; 95% CI, 1.16–3.27; *p* < 0.05), age of 70 years or older at RNU (HR, 1.67; 95% CI, 0.98–2.86; *p* = 0.06), male gender (HR, 2.51; 95% CI, 1.01–6.20; *p* < 0.05), race (other vs. white: HR, 2.53; 95% CI, 1.15–5.58; *p* < 0.05), UTUC T stage (MIUC vs. NMIUC: HR, 2.78; 95% CI, 1.46–5.31; *p* < 0.01), UTUC N stage (N1-2 vs. N0: HR, 2.42; 95% CI, 1.27–4.60; *p* < 0.01), and metastasis at RNU (M1 vs. M0: HR, 12.76; 95% CI, 5.83–27.96; *p* < 0.001) (Table 2).

**Table 2:** Uni- and multivariable competing-risk Cox regression of risk factors associated with cancer-specific survival after radical nephroureterectomy in UTUC patients with a history of radical cystectomy for bladder cancer

Variable	Class	Univariable analysis			Multivariable analysis		
		HR	95% CI	*p* value	HR	95% CI	*p* value
Age at RC (years)	≥70 vs. <70	1.950	1.163–3.271	0.0114			
Age at RNU (years)	≥70 vs. <70	1.674	0.978–2.864	0.0603			
Gender	Male vs. female	2.508	1.014–6.202	0.0465	2.356	1.025–5.416	0.0436
BC T stage	MIBC vs. NMIBC	1.071	0.576–1.992	0.8286			
	Unknown vs. NMIBC	0.979	0.532–1.801	0.9457			
BC N stage	N1-N2 vs. N0	1.826	0.396–8.417	0.4397			
	Nx vs. N0	0.979	0.571–1.678	0.9372			
LND during RC	Yes vs. no	1.327	0.629–2.797	0.4579			
M stage at RC	M1 vs. M0	1.536	0.287–8.231	0.6162			
	Mx vs. M0	0.960	0.560–1.645	0.8814			
Chemotherapy for BC	Yes vs. no	1.399	0.739–2.650	0.3025			
UTUC T stage	MIUC vs. NMIUC	2.781	1.456–5.314	0.0020	2.201	1.132–4.279	0.0200
	Unknown vs. NMIUC	4.607	1.935–10.968	0.0006	5.348	1.277–22.400	0.0218
UTUC N stage	N1-2 vs. N0	2.419	1.272–4.601	0.0071			
	Nx vs. N0	1.937	0.998–3.762	0.0508			
LND during RNU	Yes vs. no	1.326	0.794–2.216	0.2809			
M stage at RNU	M1 vs. M0	12.764	5.828–27.956	<.0001	11.591	5.332–25.199	<.0001
	Mx vs. M0	2.203	1.039–4.672	0.0393	0.617	0.134–2.854	0.5369
Chemotherapy for UTUC	Yes vs. no	1.360	0.724–2.554	0.3394			
UTUC tumor size (cm)	>2 vs. <2	1.025	0.481–2.187	0.9483			
	Unknown vs. <2	1.324	0.594–2.949	0.4921			
UTUC location	Ureter vs. pelvis	1.073	0.627–1.836	0.7978			
UTUC laterality	Right vs. left	0.838	0.498–1.410	0.5055			
Race	Black vs. white	0.746	0.182–3.048	0.6828			
	Other vs. white	2.529	1.146–5.584	0.0217			
Interval between RC and RNU (years)	<3 vs. >3	1.178	0.698–1.987	0.540			

UTUC, upper urinary tract urothelial cancer; BC, bladder cancer; HR, hazard ratio; CI, confidence interval; RC, radical cystectomy; RNU, radical nephroureterectomy; RC, radical cystectomy; MIBC, muscle-invasive bladder cancer; LND, lymph node dissection; NMIBC, non-muscle-invasive bladder cancer; MIUC, muscle-invasive urothelial cancer; NMIUC, non-muscle-invasive urothelial cancer

In univariable Cox regression analyses, the following factors were associated with OS: age of 70 years or older at RC (HR, 2.07; 95% CI, 1.28–3.34; *p* < 0.01), age of 70 years or older at RNU (HR, 2.20; 95% CI, 1.35–3.58; *p* < 0.01), male gender (HR, 2.25; 95% CI, 1.04–4.90; *p* < 0.05), metastasis at RC (M1 vs. M0: HR, 4.38; 95% CI, 1.82–10.58; *p* = 0.001); UTUC T stage (MIUC vs. NMIUC: HR, 2.85; 95% CI, 1.66–4.90; *p* < 0.001), UTUC N stage (N1-2 vs. N0: HR, 1.82; 95% CI, 1.00–3.31; *p* = 0.05), metastasis at RNU (M1 vs. M0: HR, 8.62; 95% CI, 4.11–18.08; *p* < 0.0001), and interval between bladder cancer and UTUC diagnosis (<3 vs. >3 years: HR, 1.56; 95% CI, 0.99–2.46; *p* = 0.056) (Table 3).

**Table 3:** Uni- and multivariable Cox regression analyses of risk factors associated with overall survival after radical nephroureterectomy in UTUC patients with a history of radical cystectomy for bladder cancer

Variable	Class	Univariable analysis			Multivariable analysis		
		HR	95% CI	*p* value	HR	95% CI	*p* value
Age at RC (years)	≥70 vs. <70	2.068	1.282–3.335	0.0029			
Age at RNU (years)	≥70 vs. <70	2.199	1.349–3.583	0.0016	2.212	1.249–3.917	0.0065
Gender	Male vs. female	2.254	1.038–4.894	0.0399	2.641	1.142–6.111	0.0233
BC T stage	MIBC vs. NMIBC	1.435	0.832–2.476	0.1938			
	Unknown vs. NMIBC	0.928	0.509–1.691	0.8061			
BC N stage	N1-N2 vs. N0	1.122	0.212–5.940	0.8921			
	Nx vs. N0	0.787	0.465–1.333	0.3728			
LND during RC	yes vs. no	0.647	0.363–1.152	0.1391			
M stage at RC	M1 vs. M0	4.383	1.816–10.577	0.0010	9.327	1.914–45.454	0.0057
	Mx vs. M0	0.808	0.474–1.376	0.4320	0.532	0.265–1.071	0.0772
Chemotherapy for BC	Yes vs. no	1.088	0.574–2.065	0.7957			
UTUC T stage	MIUC vs. NMIUC	2.850	1.658–4.899	0.0002	3.293	1.729–6.271	0.0003
	Unknown vs. NMIUC	2.891	1.141–7.322	0.0252	2.167	0.612–7.676	0.2309
UTUC N stage	N1-2 vs. N0	1.817	0.998–3.311	0.0509			
	Nx vs. N0	1.305	0.649–2.625	0.4556			
LND during RNU	Yes vs. no	1.082	0.686–1.707	0.7341			
M stage at RNU	M1 vs. M0	8.622	4.111–18.084	<.0001	7.875	2.919–21.245	<.0001
	Mx vs. M0	1.384	0.565–3.390	0.4774	0.605	0.109–3.359	0.5651
Chemotherapy for UTUC	Yes vs. no	0.894	0.468–1.710	0.7354			
UTUC tumor size (cm)	>2 vs. <2	1.108	0.547–2.245	0.7759			
	Unknown vs. < 2 cm	0.856	0.385–1.903	0.7023			
UTUC location	Ureter vs. pelvis	0.940	0.576–1.533	0.8034			
UTUC laterality	Right vs. left	0.753	0.475–1.195	0.2285			
Race	Black vs. white	0.809	0.302–2.168	0.6733			
	Other vs. white	1.804	0.778–4.184	0.1692			
Interval between RC and RNU (years)	<3 vs. >3	1.561	0.989–2.464	0.056	2.710	1.511–4.862	0.0008

UTUC, upper urinary tract urothelial cancer; BC, bladder cancer; HR, hazard ratio; CI, confidence interval; RNU, radical nephroureterectomy; RC, radical cystectomy; MIBC, muscle-invasive bladder cancer; NMIBC, non-muscle-invasive bladder cancer; LND, lymph node dissection; MIUC, muscle-invasive urothelial cancer; NMIUC, non-muscle-invasive urothelial cancer

### Multivariable Analysis for CSS and OS After RNU

In the multivariable competing-risk Cox regression analysis, the factors predictive for cancer-specific mortality after RNU were male gender (HR, 2.36; 95% CI, 1.03–5.42; *p* < 0.05), stage at RNU (MIUC vs. NMIUC: HR, 2.20; 95% CI, 1.13–4.28; *p* < 0.05), and presence of distant metastasis at RNU (M1 vs. M0: HR, 11.59; 95% CI, 5.33–25.2; *p* < 0.001) (Table 2).

In the multivariable Cox regression analysis, the factors predictive for overall mortality after RNU were age older than 70 years at RNU (HR, 2.21; 95% CI, 1.25–3.92; *p* < 0.01), gender (male vs. female: HR, 2.64; 95% CI, 1.14–6.11; *p* < 0.05) disease stage at RNU (MIUC vs. NMIUC: HR, 3.29; 95% CI, 1.73–6.27; *p* < 0.001), presence of distant metastasis at RNU (M1 vs. M0: HR, 7.88; 95% CI, 2.92–21.25; *p* < 0.001), presence of distant metastasis at RC (M1 vs. M0: HR, 9.33; 95% CI, 1.91–45.5; *p* < 0.001), and interval between bladder cancer and UTUC diagnosis (<3 vs. >3 years: HR, 2.71; 95% CI, 1.51–4.86; *p* < 0.001) (Table 3).

### Survival Outcomes After RC with Subsequent RNU Compared with RC Alone

To evaluate whether UTUC recurrence impairs survival outcomes after RC, CSS (Fig. 1A) and OS (Fig. 1B) were compared between risk-stratified, PS-matched patients who underwent RNU for metachronous UTUC after RC and their counterparts treated with RC alone. In the Kaplan–Meier analyses of PS-matched cohorts, UTUC requiring RNU after RC compared with no RNU was initially not associated with worse OS (*p* = 0.81) but with worse CSS (*p* = 0.02). Additional conditional survival analyses from the landmark point of 12 and 36 months after RC, showed a worse CSS (both *p* < 0.001) for the patients who had developed UTUC. A negative impact of UTUC on OS was observed in the 36-month landmark analysis (*p* = 0.02).

## Discussion

This study analyzed the survival outcomes and their predictors in patients who underwent RNU for metachronous UTUC after previous RC for bladder cancer. First, we observed very poor survival after RNU due to metachronous UTUC recurrence in patients previously treated with RC. Half of the patients succumbed within 2 years after RNU, with the majority of deaths attributable to cancer-specific causes. Second, although the majority of the patients had organ-confined bladder cancer at the time of RC, they presented with muscle-invasive UTUC at the time of RNU. Third, most often, RNU for UTUC recurrence was a late event after RC. The interval between surgeries exceeded 3 years for the majority of the patients. Furthermore, RNU was associated with a non-negligible, relatively high 3-month all-cause mortality rate of 10%. Fourth, in the PS-matched, risk-stratified cohort of bladder cancer patients treated with RC, RNU for UTUC was associated with worse CSS, and a landmark analysis at 3 years time point, showed a detrimental effect on OS. Fifth, the independent risk factors associated with cancer-specific mortality after RNU were distant metastases at RNU, UTUC T stage, and male gender.

Pan-urothelial cancer with metachronous recurrence in the UUT after RC constitutes an aggressive disease with unsatisfactory treatment outcomes.^4^ In our observation, almost two thirds of patients following RNU after RC succumb to urothelial cancer within 5 years, and 3 of 4 patients die from all causes within 5 years. Therefore, radical surgical treatment does not provide satisfactory cancer control for UTUC in that setting, and our findings align with previous studies with smaller sample sizes.^7,8^ Poor survival after RNU in patients previously treated with RC had been reported in only a few retrospective studies with small cohorts.^4,7,8^

We observed a relatively high frequency of UTUCs with adverse pathologic features at the time of RNU, with a predominance of pT3-pT4 stage disease (48% of patients) and a substantial proportion of lymph node-positive disease (22.6%). Additionally, we noted an underutilization of LND (only 49%) and perioperative chemotherapy (19.6%) at the time of RNU. High rates of locally advanced UTUCs after RC might be the consequence of suboptimal guideline adherence regarding intensity of imaging follow-up evaluation, use of non-contrast imaging due to impaired renal function with suboptimal sensitivity for UTUC detection, unsatisfactory accuracy of urine cytology, and finally, relatively late occurrence of UTUC after RC when intensity of surveillance has already been deescalated.

Perhaps all the aforementioned diagnostic methods seem not to provide a satisfactory sensitivity for metachronous UC detection.^4,6^ Other studies also have included a significant percentage of locally advanced UTUCs (pT3-pT4) and LN-positive disease at diagnosis.^5,7^ Notably, for cystectomized patients, UTUC is most commonly diagnosed due to symptoms including hematuria, flank pain, and pyelonephritis, which are already associated with advanced overlooked disease.^5^ A meta-analysis reported that 64% of patients with UC recurrence in the UUT experienced symptoms.^6^ These underscore the limitations of current follow-up schedules. Consequently, the majority of RNUs in our study were performed due to muscle-invasive and locally advanced UTUC, indicating the overlooked early-stage cancer.

On the other hand, the detection of asymptomatic disease is associated with a 30% reduction of mortality compared with symptomatic disease.^16^ The window of opportunity for effective treatment with RNU is missed when UTUC is diagnosed at a locally advanced stage, which strongly impairs the survival.^17^

As we have shown, the independent risk factors for worse CSS were muscle-invasive stage of UTUC, presence of distant metastasis at RNU, and male gender. These factors support a surveillance strategy for long-term bladder cancer survivors aimed at early detection of UC recurrence in remnant urothelium.

Currently, oncologic surveillance after RC involves regular chest and abdominopelvic computed tomography scans to detect local or distant recurrence of UC.^2^ No consensus exists concerning the regular performance of urine cytology, including urethral washing or urethroscopy for the purpose of early detection of metachronous UC recurrence.^2,6^

Overall survival was worse for the patients with muscle-invasive UTUC or metastasis at the time of RC or RNU, for males, for patient’s older than 70 years, and for patients with an interval between RC and RNU shorter than 3 years. These indicate that early diagnosis of UTUC at the time of the localized non-muscle-invasive stage increases the chances for better survival. On the other hand, age, male gender, and UTUC development early after RC constitute non-modifiable risk factors compromising survival.

A meta-analysis by Picozzi et al.^6^ indicated that the risk of UTUC after RC ranges from 0.75% to 6.4%. Especially at high-risk for recurrence in remnant urothelium were patients with multifocal NMIBC, including carcinoma *in situ,* bladder neck tumor, involvement of the prostatic urethra, and positive urothelial margins.^4^

Interestingly, in the current study, the patients with NMIBC at RC were overrepresented in the cohort of metachronous UTUCs due to the high competing mortality observed in the MIBC cohort. Stage T1 bladder cancer at the time of radical cystectomy was the most common in our study, likely due to the extended survival of these patients, allowing the development of UTUC.^18^ Notably, the indications for RC in the NMIBC setting include BCG-unresponsive disease, tumors not amenable to complete transurethral resection, and selected cases of high- and very-high-risk tumors, which are at significant risk of cancer-specific mortality.^19^

Failure of BCG therapy is relatively common, reported for approximately 20% of contemporary cohorts at high risk for NMIBC.^20,21^ Meta-analysis showed an almost threefold higher risk of UTUC after RC in superficial versus invasive bladder cancer.^6^ We identified a relatively low number of patients with locally advanced bladder cancer and subsequent RNU for UTUC, which might be attributable to very high competing short-term bladder cancer-specific mortality in these individuals. Perhaps even in the case of early UTUC recurrence for such patients, RNU is not routinely performed due to synchronous metastatic progression of bladder cancer. On the other hand, a significant proportion of patients with locally advanced bladder cancer at RC die due to bladder cancer recurrence and metastasis and do not live long enough for UTUC to develop.

Another important finding of our study was the late recurrence of metachronous UTUC after RC. The majority of UTUCs occur 3 years after RC, and within that period, the significant percentage of cystectomized patients with pT3-pT4 bladder cancer experience progression and decease.^4,5^ In our study, the median interval between RC and RNU was 49 months, which seems to be consistent with a meta-analysis reporting a median of 43 months between RC and UTUC recurrence.^5^

The reported poor survival following RNU after RC underscores the need for improvement. Administration of adjuvant chemotherapy and performance of LND as an integral part of RNU have been shown to prolong survival in UTUC, but both were underused in the studied cohort.^22-24^ Notably, the timing of the UTUC diagnosis seemed to have the greatest impact on survival, highlighting the importance of ongoing surveillance for long-term RC survivors.

In the initial analysis of the PS-matched cohort of RC patients, those with subsequent RNU for UTUC had worse CSS but not OS compared with the individuals without UTUC requiring RNU. An additional landmark analysis with 12- and 36-month time points demonstrated a worse CSS for the patients who experienced the development of metachronous UTUC.

Analysis of conditional survival among patients alive 36 months after RC showed that further development of UTUC resulted in worse OS. These results confirm the detrimental effect of UTUC on both CSS and OS. Performing RNU after previous RC can be a technically challenging surgery that requires a high level of surgical skills due to the potential complications related to urinary diversion and the post-RC anatomic and functional changes of the urinary tract. The difficulty of RNU might be dependent on the type of urinary diversion and the UTUC stage.

The perioperative all-cause mortality reported in our study was high, exceeding 10% within 3 months after surgery. This is a significantly higher rate than that observed in the primary RNU setting. Our results can help clinicians in counseling long-term RC survivors at risk of late recurrence in the UUT.

The limitations of our study included its retrospective character and missing baseline data for some patients, as reported in Table 1. On the other hand, regarding the uniqueness of the cohort, the population-based character of the study enabled us to gather a sufficient number of patients for the analysis. Selection bias was inevitable due to the inclusion of patients who survived bladder cancer and lived long enough to experience the development of metachronous UTUC. In unrestricted survival analysis, the lack of difference in OS between those who received RNU after RC and those who underwent RC alone might be the result of selection bias. Simultaneously, it underscores the high prevalence of competing mortality causes in patients after RC. However, conditional survival with a 36-month landmark point showed a worse OS for the patients who experienced the development of UTUC during the subsequent follow-up evaluation.

It is noteworthy that in our cohort, perioperative chemotherapy for UTUC did not influence survival, but the absence of data on the regimen and number of cycles constituted a major confounder. The low utilization of chemotherapy in this cohort may have been attributable to the high morbidity burden and impaired renal function in a significant proportion of the patients. The absence of data regarding the status of surgical margins may be considered a limitation in the risk factor analysis.

Another limitation of our study was the lack of available data regarding the curative intent of the surgery for the analyzed patients. It is important to note that some of the patients may have undergone RNU due to symptoms or as a palliative procedure. The inclusion of a proportion of patients with metastatic UTUC (5.8% M1 and 4.9% Mx) and the observation of poor median overall survival after RNU suggest that at least some of the surgeries may not have been performed with a curative intent. This limitation raises the possibility that our findings could have been influenced by non-curative surgeries, emphasizing the need to interpret the results with caution in light of this uncertainty. On the other hand, it is worth noting that long-term survivors also were identified, with 25% of the cohort surviving more than 5 years afterward, which is evidence for disease cure. Reliable survival data and relatively long-term follow-up evaluation must be considered as strengths of the study.

## Conclusion

In conclusion, patients who underwent RNU for UTUC after RC for bladder cancer experienced poor overall and cancer-specific survival. Metachronous UTUC recurrence is a late oncologic event after RC, placing long-term RC survivors at risk. The majority of RNUs are performed for muscle-invasive UTUC, which constitutes an adverse prognostic factor.

## Data Availability

The datasets analyzed during the current study are available from the U.S. National Cancer Institute upon request.
